# The relationship of three-dimensional foot morphology to clinical assessments and postural stability in adolescent male footballers

**DOI:** 10.1186/s13047-023-00636-w

**Published:** 2023-08-19

**Authors:** Ferdia Fallon Verbruggen, Jitka Marenčáková, František Zahálka

**Affiliations:** Sport Research Centre, Faculty of Physical Education and Sport, Charles University, José Martího 269/31, Praha 6, 162 00 Prague, Czech Republic

**Keywords:** Foot morphology, Principal component analysis, Postural stability, Adolescents, Football, Three-dimensional shape

## Abstract

**Background:**

Foot morphology is associated with altered loading of the ankle–foot complex in adolescent footballers, predisposing to pain and injury. However, usual singular plane clinical assessments do not accurately capture the 3D nature of foot morphology. A new approach is 3D laser scanning, with statistical shape model techniques creating individual-to-group comparison. However, no research exists on the adolescent, football-playing foot. Furthermore, a link between 3D foot morphology, and usual clinical and performance measures would be beneficial for practical implementation.

**Methods:**

Four hundred forty-seven 3D foot scans from 224 elite male footballers (U12-U19) in bilateral stance were collected and further processed with statistical shape model techniques. Weighted shape parameters for individual principal components (Modes) were extracted for each foot. Centre of pressure displacement expressed as total travelled way in millimetres was calculated for bilateral and unilateral postural stability measures. Clinical assessments (Clarke’s Angle, Resting Calcaneal Stance Position) were calculated on the 3D foot scans. Differences in weighted shape parameters, postural stability measures, and clinical assessments between age groups were determined by ANOVA. Correlations determined the relationship of Modes and clinical assessments to postural stability measures. Linear regression established if clinical assessments predicted the mode describing foot arch variation.

**Results:**

Age groups significantly differed for Mode 1 (foot length), Mode 2 (foot arch), and Mode 5 (tibial rotation relative to the foot) (*p* < 0.05). Resting Calcaneal Stance Position (*r* = .663) and Clarke’s Angle (*r* = -.445) were low-to-moderately correlated to Mode 2 (both *p* < 0.001), and linear regression found they were both significant predictors of Mode 2, though only moderately (R^2^ = .522). There were low correlations of foot morphology to the postural stability tests.

**Conclusion:**

This is the first study to describe the 3D foot morphology of male football-playing adolescents, and discover the differences between age groups. This will improve understanding and assessment of foot morphology in male adolescents because 2D techniques, as discovered in this study, do not strongly correlate to, nor predict, the 3D foot arch. Foot morphology was only lowly correlated to postural stability, thus a multifaceted program would be required for improvements.

## Background

Foot and ankle injuries are highly prevalent in adolescents [1–3]. This is especially true for footballers, who have an increased risk of fractures and epiphyseal injuries [1–3]. These injuries can lead to long periods of physical inactivity, which can have a detrimental effect on a patient’s quality of life [4]. One factor that can alter loading of the ankle–foot complex, and potentially lead to pain and injury, is foot morphology.

The morphology of the foot has been well researched in order to establish its link to increased pain and injury in the foot and ankle [5]. The foot arch has received the most focus because a flat arch (pes planus) or a high arch (pes cavus) presentation can lead to altered loading of the foot during athletic tasks, and decreased static and dynamic postural stability performance [6–9]. Altered loading from foot morphology is a particular concern in adolescents, where it could further overload and lead to significant foot fractures and overuse injuries [10–12]. Thus, foot morphology assessment is warranted as a routine part of football screening.

Foot morphology assessment is usually carried out in a clinical setting because medical imaging is costly, time-consuming, and any potential radiation risk would be unsuitable for a young, asymptomatic population, as per ALARA principles [13]. While various assessments exist, few have been validated in the adolescent population [14]. Clarke’s Angle has been recently confirmed as reliable and valid in adolescents of all ages [14–16], while Resting Calcaneal Stance Position is strongly correlated with imaging methods, and it has been used to define cut-offs in adolescents previously [17, 18]. However, a limitation of these analyses is that they are two-dimensional (2D) approaches to the three-dimensional (3D) foot, missing the global picture from forefoot to hindfoot [19]. While whole foot approaches like the Foot Posture Index 6 (FPI-6) exist, they can lack reliability due to their subjective assessment [16, 20]. While readily accessible, these limitations to current 2D and/or subjective clinical assessments make objective 3D approaches attractive for accurate diagnosis and treatment for those in large health centres or in sporting environments, where more funding for equipment is available and large populations are tested more frequently. Moreover, the costs are constantly decreasing with models available from approximately $200USD on the market [21].

The most common 3D approach to foot assessment is laser scanning. Laser scanning has no risk of radiation, and offers a fast, reliable, and accurate assessment [22]. However, a limitation is the interpretation of the foot scans in regards to the population being compared against. This normative population description can be defined using Statistical Shape Models (SSMs). Using 3D foot scanning technology, three studies have attempted to define normative data values for adult populations [23–25]. Mei et al. analysed the difference between habitually barefoot and habitually shod males, and they found a variance in principal component generation, though specific differences between groups were not determined [24]. Conrad et al. had a large sample size of over 1700 females and 2400 males, and they discovered that females had a higher arch and instep, as well as a narrower foot compared to males. However, the paper lacked elaboration and the foot shape did not specify toe morphology [23]. Stanković et al. (2018) described in-detail normative data for a healthy adult population with a specific foot shape that showcased the intricacies of each principal component that related to arch height, forefoot type, heel variation, hallux angulation, and midfoot width, amongst others. They found significant results in relation to gender, age, and shoe size, and showed individual foot comparisons to the population [26]. This study group additionally validated the same techniques in analysing abnormal foot arches and hallux variation, and could completely characterise these abnormalities in 3D for the first time [25]. However, such SSM analysis has not been conducted in an adolescent, football-playing population, despite the repetitive loading of the foot and ankle complex and potential injury risk associated with foot morphology.

Thus, the first aim of this research was to describe 3D foot morphology differences between age groups of elite male adolescent footballers. Furthermore, it would be beneficial to understand how much usual clinical measures can relate to and explain the 3D foot presentation. Thus, the second aim was to compare clinical assessments to the specific foot morphology related to arch height in this population by correlation and linear regression analyses. Since this is a football-playing population, to discover the relationship between foot morphology and a performance-related measure would be beneficial for sport practitioners. Therefore, the last aim of this research was to assess the relationship between foot morphology and postural stability measures.

## Methods

### Study sample and recruitment

This was a retrospective, cross-sectional study. The U12-U19 squads of two elite football academies in the Czech Republic, that presented for pre-, mid-, or post-season testing in one season, were analysed for this study. This testing was conducted at the Sport Research Centre at the Faculty of Physical Education and Sports, Charles University. Players had proof of a signed contract to train and play with their club and gave informed consent for the testing by themselves or with parents/guardians if underage. Lower limb injury or pain, which would affect the ability to weightbear, ankle brace use, or orthoses use were exclusion criteria. The most recent testing timepoint for a player was selected, and players between the ages of 10.5 and 18.5 were included in the study. The half point is because, while the season runs from summer to summer in the Czech Republic, the cut-off point for age groups is the first of January of each year. This study was approved by the ethics committee of the Faculty of Physical Education and Sport at Charles University (Czech Republic), under approval No. 107/2021.

### Sample size

 Two hundred twenty-four players were analysed for this research. To counteract confounding factors of players playing up or down age groups, as well as the combined age group of U18-19 in Czech football academies, players were stratified into age group by their year of birth for the period of time that they were tested. The number of participants per age group are included in Table 1.

With left and right foot analysed, 448 foot scans were collected. Due to a field of view error in scanning, 1 scan from the U17 group was excluded from the trial. The 447 foot scans remaining are still above the recommended 200 population sample size recommended in the literature for statistical shape model purposes [27].

## Testing protocol

### Baseline characteristics

Baseline data gathered were date of birth, date of measurement, height, weight, and shoe size. Shoe size was reported in the European form. Weight was measured by the Tanita MC-980 MA Plus (Tanita Corporation, Toyko, Japan), while standing height was measured with a Seca 213 stadiometer (Seca, Hamburg, Germany).

### Foot scan statistical shape models

Both feet were scanned in bilateral stance on the Elinvision Tiger 3D laser scanner (RS scan, Belgium). Players stood barefoot with feet hip-width apart, hands on hips, and head facing forward. The second toe and posterior ankle were in line with the middle of the scanner to minimise rotation of the lower limb, which can have an impact on foot posture [28]. This has been previously reported in the literature [26], and weightbearing has been recommended over non-weight bearing measures due to increased accuracy and consistency [13].

To create the statistical shape models (SSMs), foot scans were exported as stereolithography (STL) files and cropped at 2.0 cm above the malleolus [26]. Left feet were mirrored to pseudo right feet in order to generate the foot model [29]. Each STL file was re-orientated to a consistent XYZ coordinate system using an interactive Python tool, and transformed to an arbitrary local position representative of the average of all STL points for that given foot.

Transformed STL files were processed through an established SSMs framework [29, 30], to generate a SSM of the foot for the entire population: First, meshes from one subject were non-rigidly fit to all the remaining segmentations to ensure mesh and node correspondence. Second, all meshes were rigidly aligned to remove global rotational and translational variations. The final step factorised the aligned meshes using principal component (PC) analysis to extract the mean shape and the primary shape variations in the form of principal components (Modes).

Modes were extracted for the foot along with each individual subject’s weighted shape parameter for each Mode, indicating how far a subject’s geometry is from the average for a specific Mode. For each comparison of the Modes for the foot, surface distances were calculated between the mean shape and those representing ± 2 standard deviations (SD) from the mean, and visualised using CloudCompare (Version 2.11, EDF R&D, France).

### Clinical assessments

Foot Length and Foot Width were calculated as per IEEE standards [31]. Foot Length was calculated by the Acropodion, or ‘furthest toe’, to the Pternion, or ‘the centre of the back of the heel’ [31]. Foot Width was measured between the Metatarsale tibiale, ‘first metatarsal’, and the metatarsale fibulare, ‘fifth metatarsal’ [31].

In order to compare the 3D morphology to usual clinical practice, two assessments were calculated on the 3D foot scans: Resting Calcaneal Stance Position and Clarke’s Angle. These two tests are validated in the adolescent population and do not require palpation of the individual [14, 16, 17]. They also represent different areas of the foot, are thoroughly described in the literature, and are possible to calculate on 3D foot scans [14].

Clarke’s Angle: It was shown to have higher inter- and intra-rater reliability, and accuracy than FPI-6 [16]. As per that study, Clarke’s Angle was obtained by calculating the angle between the medial tangential line, connecting the medial edges of the first metatarsal head and the heel, and a second line connecting the first metatarsal head and the acme of the medial longitudinal arch concavity.

Resting Calcaneal Stance Position: It is measured in bilateral stance, and correlates well with hindfoot angles taken from radiographic measurements [32]. The guidelines from de Cesar Netto et al. were adapted for this research [32]. A correction was made in order to make this two-dimensional clinical measure more accurate for the three-dimensional foot. Points were placed on the lateral and medial malleoli, and a line was connected between them. This was also done for the most medial and lateral aspects of the heel. A line connecting the midpoints and a vertical line through the malleoli midpoint then created the RCSP angle.

The IEEE standards do not include measurements for these two assessments in their whitepaper [31]. These calculations were created by placing points on the 3D foot scans using 3D Slicer (Brigham and Women’s Hospital, USA) (Appendix 1). These points were then exported as an .npy file, and calculations of the angles were completed using an interactive Python tool.

### Postural stability

The postural stability tests selected are those detailed in Marencakova et al. (2018). These tests were also conducted in youth footballers, and they used the same force platform (RS scan, Belgium) and software (FootBalance 7, rs scan, Belgium) as this present study [33]. With shoes and socks off, strict adherence to foot posture placement was followed to standardise results, and researchers ensured the feet faced forward and were equally placed on the force platform. Firstly, the calm narrow standing test was performed, one trial with eyes open (Bilateral Open Eyes), followed by a trial with eyes closed (Bilateral Closed Eyes). These were captured at 33 frames per second for 30 s. Then both legs were measured in unilateral, flamingo stance with eyes open (Unilateral Open Eyes). These were captured at 17 frames per second for 59 s. Centre of pressure displacement expressed as total travelled way in millimetres was calculated for each test.

### Data analysis

Data was collected and analysed with SPSS (Version 25, IBM, USA). The data was normally distributed for all parameters. Descriptive statistics described baseline statistics, postural stability results, and clinical assessment results for each age group. Bayesian information criterion was used to select Modes that described the most variance in the foot morphology of the adolescent population. The average weighted shape parameters for each Mode were compared between age groups by ANOVA, with Bonferroni post hoc analysis applied. Correlation analysis determined the relationship of each mode and clinical assessment to each of the postural stability measures, as well as the relationship of the two clinical assessments to the mode describing the foot arch, with criteria set as: Negligible: 0.00 ≥ r < 0.30, Low: 0.30 ≥ r < 0.50, Moderate: 0.50 ≥ r < 0.70, High: 0.70 ≥ r < 0.90, and Very High: 0.90 ≥ r ≤ 1.00 [34]. Linear regression was run to establish the prediction of the mode that describes the foot arch variation by the usual clinical assessments. For all data analysis, significance was set at *p* < 0.05.

## Results

### Baseline characteristics 

Baseline characteristics for each age group are presented in Table 1. Significant differences were found for Age between all groups (*p* < 0.001), Height between all groups except for U17 v U16/U18/U19, and U18 v U19 (all *p* < 0.05), and Weight between all groups except for U13 v U14 and U18 v U19 (all *p* < 0.05). Shoe Size, Foot Length, and Foot Width were significantly different between groups, (*p* < 0.05), except between U13 v U14 (all three parameters), U14 v U15 (Foot Length), U15 v U16 (Foot Length/Width), and between the U16-U19 age groups (all three parameters).

**Table 1:** Means and standard deviations of baseline characteristics for the U12-U19 age groups

	**U12 (*n* = 23)**	**U13 (*n* = 26)**	**U14 (*n* = 21)**	**U15 (*n* = 30)**	**U16 (*n* = 28)**	**U17 (*n* = 42)**	**U18 (*n* = 33)**	**U19 (*n* = 21)**
**Age [years] (± SD)**	11.25 (0.238)	12.35 (0.246)	13.26 (0.288)	14.33 (0.325)	15.18 (0.266)	16.19 (0.259)	17.13 (0.310)	18.13 (0.260)
**Height [cm] (± SD)**	149.44 (6.17)	155.09 (5.79)	161.68 (5.99)	170.79 (7.60)	176.54 (6.58)	178.94 (6.23)	182.18 (5.73)	181.61 (8.27)
**Weight [kg] (± SD)**	37.27 (4.54)	43.40 (5.71)	47.51 (4.12)	56.03 (8.31)	62.88 (7.78)	68.34 (7.55)	72.57 (6.72)	75.15 (8.09)
**Shoe Size [EU] (± SD)**	37.50 (1.75)	39.80 (2.05)	40.70 (1.98)	42.10 (1.72)	43.30 (1.67)	43.60 (1.58)	43.80 (1.50)	44.20 (2.17)
**Foot Length [cm] (± SD)**	23.67 (1.10)	24.67 (1.37)	25.36 (1.44)	25.87 (1.13)	26.47 (0.95)	26.86 (1.09)	26.77 (1.26)	27.23 (1.89)
**Foot Width [cm] (± SD)**	8.91 (0.40)	9.34 (0.47)	9.67 (0.49)	10.01 (0.50)	10.19 (0.50)	10.30 (0.56)	10.32 (0.55)	10.41 (0.57)

*Legend*: *SD* Standard deviation, *cm* centimetres, *kg* kilograms, *EU* European shoe size

### Statistical shape model

**Fig. 1:**
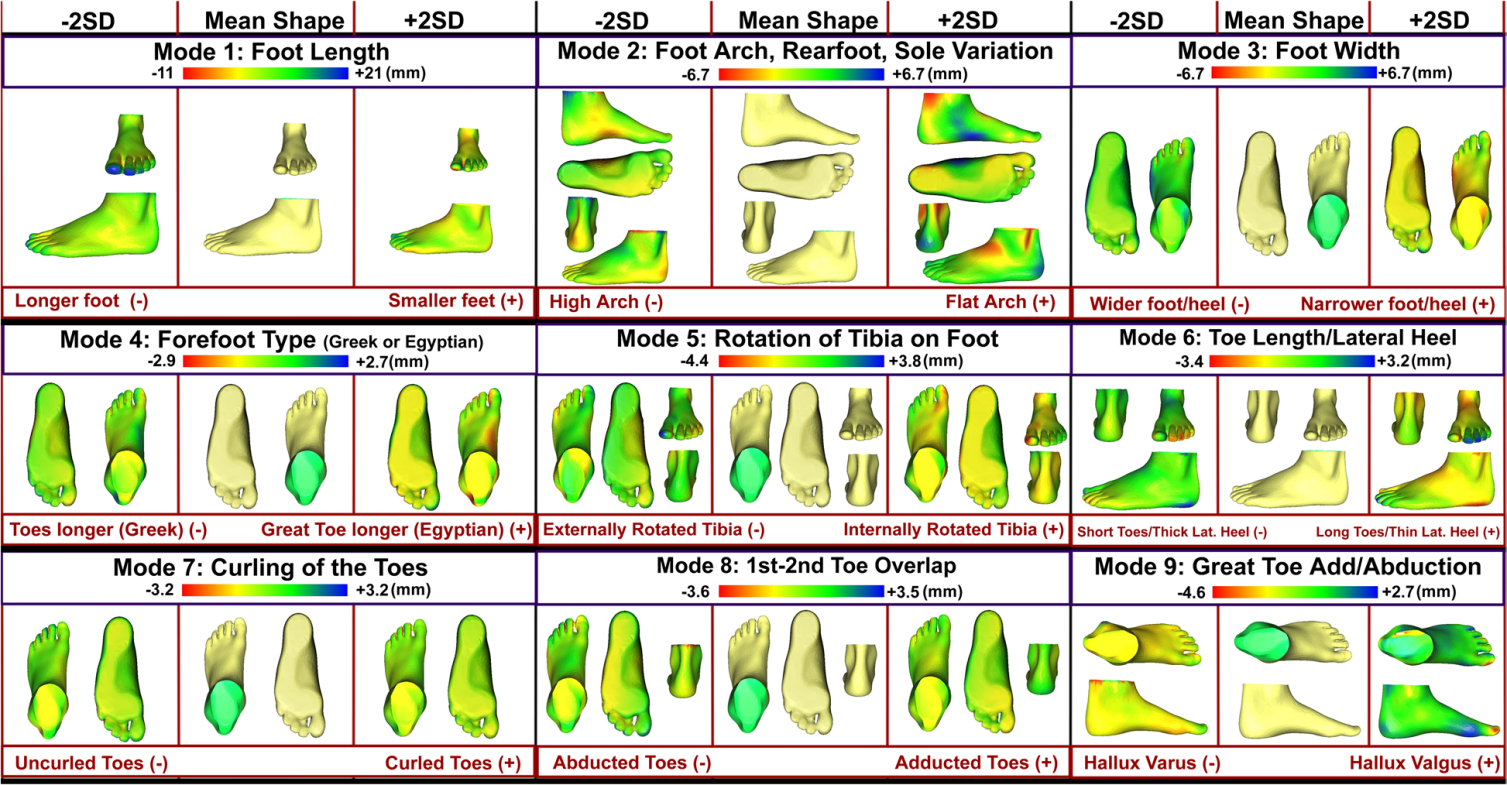
The first nine modes that described the most variance in foot shape, and their respective changes of foot shape in the direction of ± 2 standard deviations from the mean shape on a millimetre (mm) scale

The first nine PC modes of the foot, in order of decreasing variance explained approximately 77.22% of total foot shape variation (Appendix 2). The shape variations identified are described in Fig. 1. Each mode is displayed as ± 2SD compared to the mean shape. Interpretation is illustrated as the effect of the mode on the mean shape in the positive and negative direction. Mode 1 refers to the length of the foot (- longer foot, + smaller foot), and Mode 2 refers to the arch of the foot with rearfoot, sole, and arch height variation (- high arched foot, + flat arched foot). Mode 3 describes the width of the foot (- wider heel and foot, + narrower heel and foot), while Mode 4 describes the forefoot type based on whether the great toe is longer than the following toes (- Greek foot/toes longer, + Egyptian foot/great toe longer). Mode 5 describes the rotation of the tibia and its effect on the foot. A longer medial side of the foot is present when the tibia is externally rotated, and a longer lateral side of the foot is present when the tibia is more internally rotated (- externally rotated tibia, + internally rotated tibia). Mode 6 describes the length of the toes and thickness of the lateral heel (- shorter toes/thicker lateral heel, + longer toes/thinner lateral heel), and Mode 7 describes curling of the toes (- uncurled, + curled). Finally, Mode 8 describes the adduction and abduction of the toes (- abducted toes, + adducted toes), while Mode 9 describes the hallux variation (- hallux varus, + hallux valgus).

### Differences of modes between age groups

**Fig. 2:**
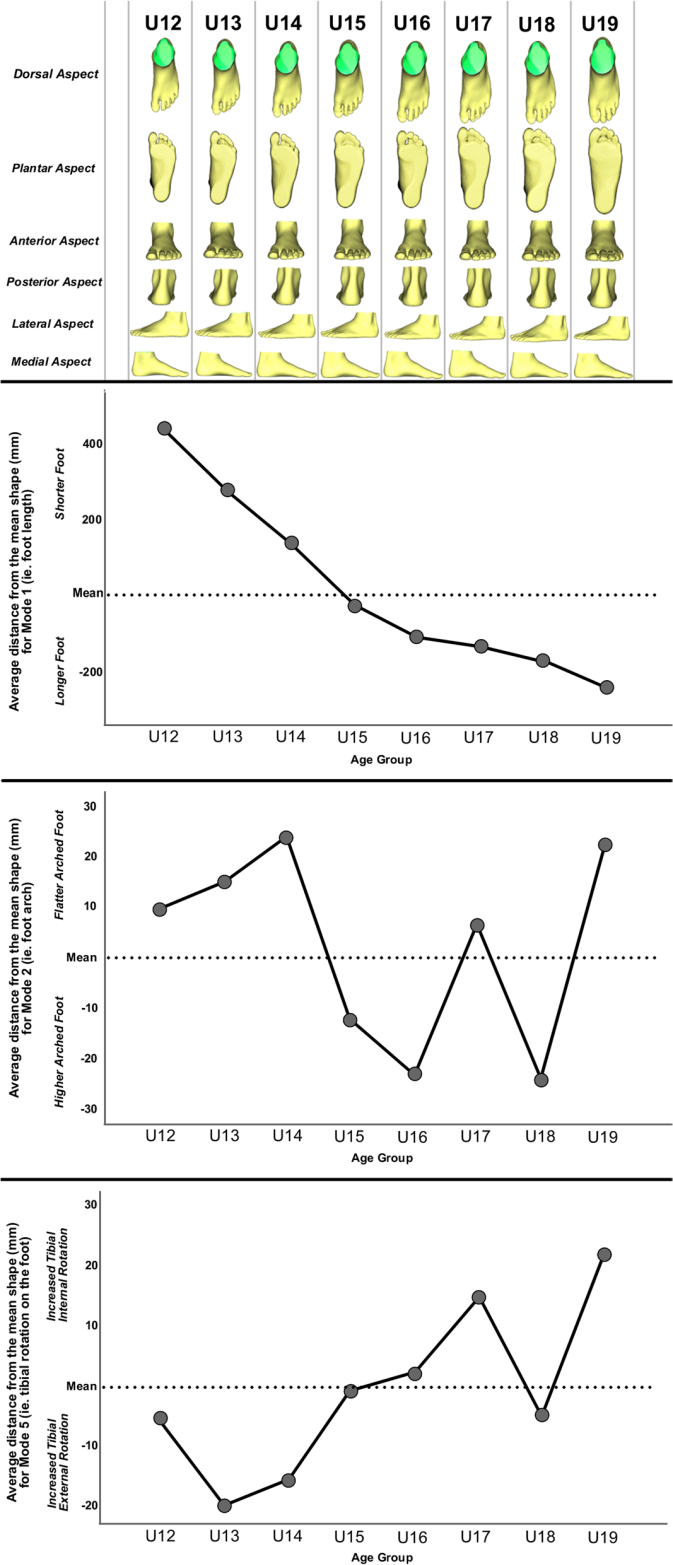
Foot models for each age group are presented at the top, followed by graphs of the age group means for Mode 1 (ie. foot length), Mode 2 (ie. flat arch or high arch), and Mode 5 (ie. rotation of the tibia relative to the foot)

One-way ANOVA revealed significant differences between groups only for Mode 1 [F(7, 446) = 69.187, *p* < 0.001], Mode 2 [F(7, 446) = 4.794, *p* < 0.001], Mode 4 [F(7, 446 = 2.246, *p* = 0.030], and Mode 5 [F(7, 446) = 5.566, *p* < 0.001]. For Mode 1 (i.e. foot length), Bonferroni post hoc analysis showed all groups significantly differed, except for U15 v U16 (*p* = 0.506), the U16-U18 squads between each other (*p* = 1.000), and U17/U18 v U19 (*p* = 1.000). For Mode 2 (i.e. flat foot or high arched), Bonferroni post hoc analysis found significance between U13 v U18 (*p* = 0.035), U14 v U16 (*p* = 0.012), U14 v U18 (*p* = 0.006), U16 v U19 (*p* = 0.019), and U18 v U19 (*p* = 0.010). For Mode 4 (i.e. Egyptian or Greek forefoot type), Bonferroni post hoc analysis revealed no significance between groups. For Mode 5 (i.e. rotation of the tibia relative to the foot), Bonferroni post hoc analysis showed significance between U13 v U17 (*p* < 0.001), U13 v U19 (*p* < 0.001), U14 v U17 (*p* = 0.007), U14 v U19 (*p* = 0.002), and U18 v U19 (*p* = 0.042). The means for each age group for Mode 1, Mode 2, and Mode 5 are presented in Fig. 2.

### Comparison between modes and clinical measures

**Fig. 3:**
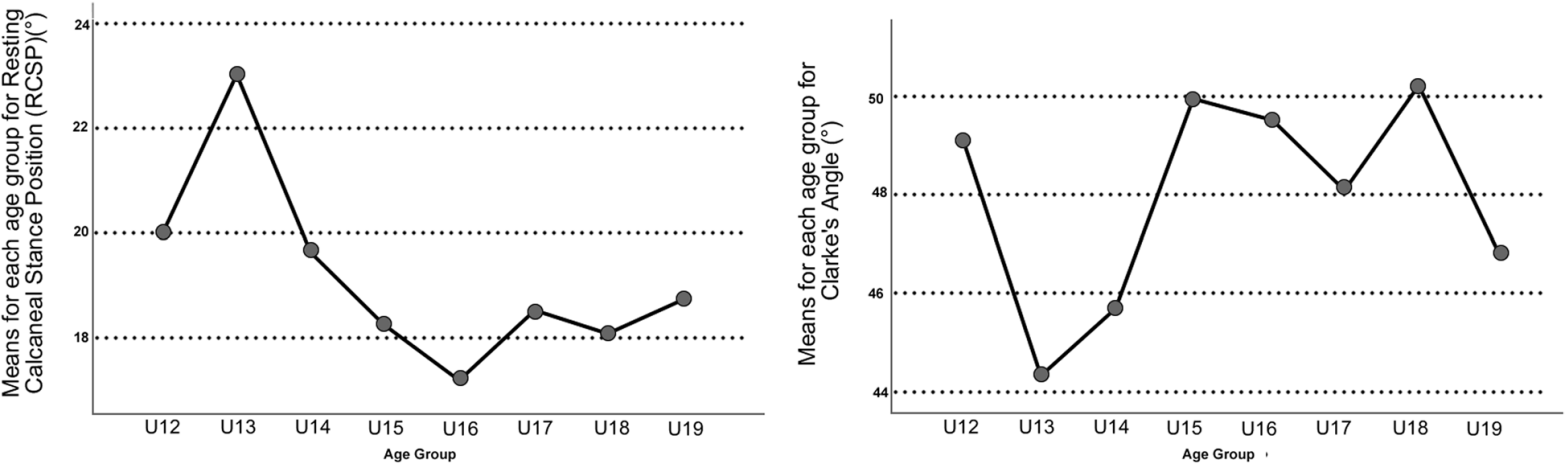
The means of each age group for Resting Calcaneal Stance Positon (Left) and Clarke’s Angle (Right)

The means and standard deviations for Resting Calcaneal Stance Positon (RCSP) and Clarke’s Angle (CA) for each age group are presented in Table 2. RCSP was significantly different between age groups, [F(7, 446) = 2.991, *p* = 0.004], with the U13 age group significantly higher compared to the U15-18 age groups on Bonferroni post hoc analysis (*p* < 0.028 for all) (Fig. 3). CA was significantly different between age groups, [F(7, 446) = 3.333, *p* = 0.002], and showed near similar results on Bonferroni post hoc analysis, with the U13 age group significantly lower compared to the U15, U16, and U18 age groups (*p* < 0.047 for all) (Fig. 3).

**Table 2:** Resting Calcaneal Stance Position and Clarke’s Angle means and standard deviations for each age group

	**U12 (*n* = 23)**	**U13 (*n* = 26)**	**U14 (*n* = 21)**	**U15 (*n* = 30)**	**U16 (*n* = 28)**	**U17 (*n* = 42)**	**U18 (*n* = 33)**	**U19 (*n* = 21)**
**RCSP(**^**o**^**) (± SD)**	20.04 (4.8)	20.41 (3.8)	19.70 (4.1)	18.27 (4.3)	17.24 (4.0)	18.50 (4.7)	18.08 (4.1)	18.76 (5.2)
**CA(**^**o**^**) (± SD)**	49.1 (9.6)	44.3 (8.7)	45.7 (10.1)	49.9 (8.9)	49.5 (7.6)	48.1 (8.0)	50.3 (7.6)	46.8 (9.3)

*Legend*: *SD* Standard deviation, *RCSP* Resting Calcaneal Stance Position, *CA* Clarke’s Angle, ^*o*^ degrees

When assessing the correlation of RCSP and CA to Mode 2 (i.e., flat foot or high arched), Pearson Correlation found that both measures significantly correlated with the mode (both *p* < 0.001). RCSP had a moderate positive correlation (*r* = 0.663), i.e., as the foot arch was flatter, RCSP increased. CA had a low negative correlation (*r* = -0.445), i.e., as the foot arch was flatter, CA decreased. The linear regression model revealed that both clinical assessments were significant predictors of Mode 2 [R^2^ = 0.522, F(2,444) = 242.244, *p* < 0.001], and the formula for the three-dimensional foot arch was *Mode 2* = *-57.065* + *8.871(RCSP) -2.272(CA)*.

### Comparison between modes, measures, and postural stability

**Fig. 4:**
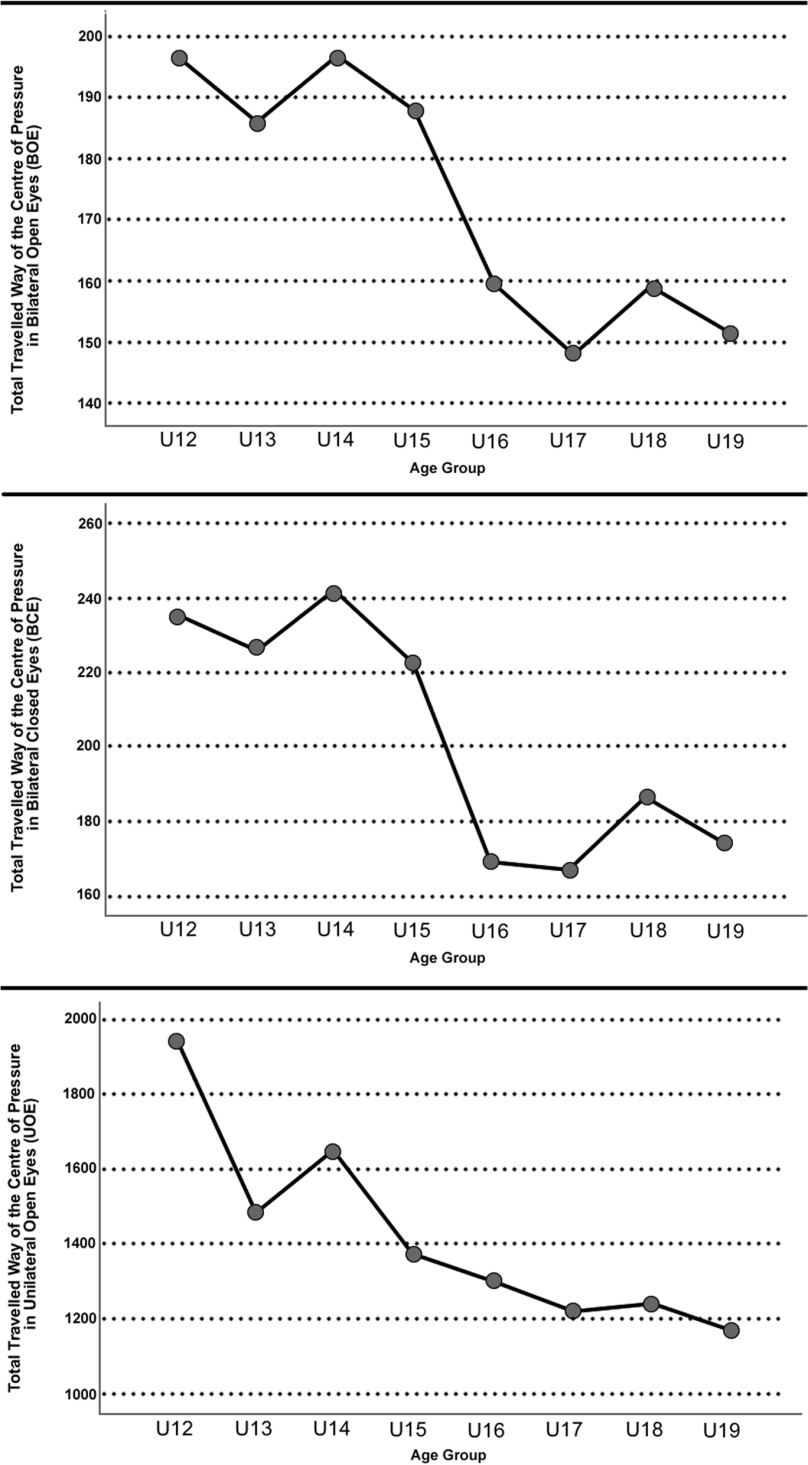
Postural stability means for each age group (Top: Bilateral Open Eyes, Centre: Bilateral Closed Eyes, Bottom: Unilateral Open Eyes)

The means for the three postural stability measures (Bilateral Open Eyes [BOE], Bilateral Closed Eyes [BCE], and Unilateral Open Eyes [UOE]) are presented in Table 3 and displayed in Fig. 4. There were significant differences between age groups for all three postural stability measures: BOE [F(7,439) = 12.967, *p* < 0.001], BCE [F(7, 439) = 11.988, *p* < 0.001], UOE[F(7, 439) = 19.806, *p* < 0.001]. For BOE, Bonferroni post hoc analysis found significant differences between U12-U15 v U16-U19 (all *p* < 0.041). For BCE, Bonferroni post hoc analysis found that there was significant differences between U12-U14 v U16-U19 (all *p* < 0.006), and U15 v U16/17/19 (all *p* < 0.010). U15 v U18 in this measure was nearly significant (*p* = 0.086). For UOE, Bonferroni post hoc analysis revealed significant differences between U12 and all other age groups (all *p* < 0.025), U13 v U17-U19 (all *p* < 0.048), and U14 v U15-U19 (all *p* < 0.034).

**Table 3:** Postural stability means and standard deviations for each age group

	**U12 (*n* = 23)**	**U13 (*n* = 26)**	**U14 (*n* = 21)**	**U15 (*n* = 30)**	**U16 (*n* = 28)**	**U17 (*n* = 42)**	**U18 (*n* = 33)**	**U19 (*n* = 21)**
**BOE (mm) (± SD)**	196.3 (45.4)	185.5 (48.7)	196.7 (46.2)	187.6 (59.3)	159.5 (33.7)	148.1 (21.4)	158.8 (24.8)	151.6 (56.2)
**BCE (mm) (± SD)**	234.9 (68.8)	226.2 (100.9)	241.6 (81.9)	222.5 (72.9)	168.7 (52.8)	166.8 (44.8)	186.7 (49.3)	173.7 (69.4)
**UOE (mm) (± SD)**	1943.4 (507.4)	1483.4 (479.6)	1648.0 (520.7)	1377.0 (385.3)	1303.5 (387.8)	1220.1 (351.5)	1242.0 (336.2)	1171.9 (374.8)

*Legend*: *SD* standard deviation, *mm* Total Travelled Way of Centre of Pressure in millimetres, *BOE* Bilateral Open Eyes, *BCE* Bilateral Closed Eyes, *UOE* Unilateral Open Eyes

When exploring the relationship between clinical assessments, baseline characteristics, and foot modes to postural stability measures, significant negligible-to-low correlations were found, with further details of their direction in Table 4. For BOE, overall there were low correlations to Age, Height, Weight, and negligible correlations to Mode 1 (i.e., foot length), Mode 2 (i.e., foot arch), Mode 4 (i.e., foot type), RCSP, and Mode 8 (i.e., toe add/abduction). For BCE, overall there were low correlations to Age, and negligible correlations to Height, Weight, Mode 1 (i.e., foot length), and Mode 5 (i.e., tibia rotation relative to the foot). For UOE, overall there were low correlations to Age, Height, and Weight, Mode 1 (i.e., foot length), and negligible correlations to Mode 3 (i.e., foot width), Mode 5 (i.e., tibia rotation relative to the foot), and Mode 8 (i.e., toes add/abduction).

**Table 4:** Correlation coefficients of age, height, weight, the nine modes of foot morphology, and clinical assessments to postural stability results

	Age	Height	Weight	Mode 1	Mode 2	Mode 3	Mode 4	Mode 5	Mode 6	Mode 7	Mode 8	Mode 9	RCSP	CA
BOE	***-.363***	***-.265***	***-.331***	***.192***	***.123***	***.179***	NS	***-.142***	NS	NS	**-.095**	NS	**.109**	NS
BCE	***-.332***	***-.256***	***-.288***	***.165***	NS	NS	NS	-.112	NS	NS	NS	NS	NS	NS
UOE	***-.444***	***-.381***	***-.420***	***.354***	NS	***.179***	NS	***-.136***	NS	NS	***-.131***	NS	NS	NS

*Legend*: *BOE* Bilateral Open Eyes, *BCE* Bilateral Closed Eyes, *UOE* Unilateral Open Eyes, *RCSP* Resting Calcaneal Stance Position, *CA* Clarke’s Angle; Level of significance, *NS* Not significant; Bold—*p* < 0.05; Bold and Italics—*p* < 0.01

## Discussion

The 3D foot SSM of male adolescent footballers detailed nine modes of foot morphology, which explained 77.22% of the foot shape variance. This is in contrast to Stanković et al. (2018), where they accounted for the variation of foot length before creating their model [26]. Thus, their first reported mode describes the foot arch, which is the second mode in this current study. Due to this difference in approach, the models should not be conflated. Previous research has shown that foot length increases over time in adolescents, and the model in this paper indicated that this plateaued at 16 years of age as shown in Mode 1 (Fig. 1) [35]. This suggests that those before and up to the age of 16 should continually monitor the length and size of their foot for correct footwear during sport to prevent discomfort from ill-fitting boots.

Mode 2 is similar to findings in Stanković et al. (2018); that the entire 3D foot arch can be captured from forefoot to hindfoot [26]. In contrast to the literature, which has found decreased flatfoot prevalence in older adolescents [14, 36], the results here show that it varied from U15 onwards, as both the U17 and U19 age groups presented with more pronated feet compared to U16 and U18. These are important findings for the literature and clinical practice, as it cannot be assumed that there is a linear pattern to foot arch presentation in adolescents. This makes foot assessment crucial for all ages to discover if they lie outside of the normal ranges for their age group, as foot type variability is considered [14]. 3D foot scans allow interpretation of these results as a global picture as opposed to usual clinical assessments, as shown by the results of our second objective.

Only low-to-moderate correlations and regression models were found when comparing the usual clinical assessments of CA and RCSP to the mode that specifies arch height (Mode 2). This indicates that these 2D measures do not capture the complete multifaceted nature of 3D foot morphology in flat or high arched feet. This is consistent with the literature, which shows that 2D measures are not as valid and reliable compared to 3D techniques [14, 37]. Interestingly, the results displayed different peaks and troughs of foot arch presentation. In both the clinical assessments, the U13 age group had the flattest foot presentation. This is in comparison to our model, which showed that the U14 age group had the ‘flattest’ 3D foot arch morphology. The differently identified ‘flattest arched’ groups and the regression analysis indicates that there is more to 3D foot arch presentation than 2D rearfoot and plantar sole analysis can determine alone.

Mode 5 showed the effect of tibial rotation on the foot, despite standardised protocols to eliminate rotational factors on scanning and in the SSM analysis. As per Schultz et al., adolescents tend to move from a pronated foot and internally rotated tibia to a supinated and externally rotated tibia [38]. Our results showed similar findings overall, especially in the U16-U19 age groups. The U16 and U18 age groups presented with a more supinated foot, as well as a more externally rotated tibia. In contrast, the U17 and U19 age groups presented with a more pronated foot, as well as a more internally rotated tibia. However, an interesting finding is that children started off in the U12 age groups with a more supinated foot too. Older age groups then had a more internally rotated tibia before the foot pronated. Following this foot pronation, the tibia presented with more external rotation. This potential coupling of the tibial torsion and foot gait mechanics, such as the Foot Progression Angle, has been well documented in the literature [39], and increased external rotation of the lower limb has been observed in those with flatfoot [40]. This may explain why a more externally rotated tibia was found in older age groups to counteract a more pronated foot presentation. Alongside further dynamic gait analysis, these findings could suggest recommendations for different age groups to prevent overload [41]. As U12 players move from a supinated to a pronated foot, potentially more external rotation strength could benefit the lower limb. Older age groups varied considerably, so their results should be interpreted individually as to whether they require more internal or external alignment interventions of the lower limb and foot.

Modes 3, 4, 6–9 were not significant in this study. Mode 3, which corresponds to foot width, surprisingly showed no significant differences between age groups, even though the literature reports a significant increase in foot width at 13–14 years of age in boys [42]. This may have been due to the wide variance of foot width in our population. Larger sample numbers, as are used in population studies, may discover a significance [42]. The other modes became more specific, and may be more related to individual abnormalities rather than age group specificities. For instance, Mode 9, corresponding to hallux abduction or adduction, was not significant between age groups, perhaps due to the fact that abnormal presentations of hallux valgus have a low prevalence of 7.8% in adolescents, and they are more common in females than males [43, 44]. However, the importance of these modes should be noted. They are the first time 3D foot morphology has been reported for adolescents, and are novel findings for the literature. They can guide better understanding of the variance of the adolescent foot, and what areas should be of focus in clinical assessment, with potential caution if significant differences are found compared to the mean shape for those non-significant modes, such as great toe length or hallux angle.

Footballers have been shown to have the second best postural stability when compared to other sports and controls in bilateral, open eyes position [45]. In our results, postural stability measures showed a trend of stabilising in the U16 group as, while they continued to decrease, they became non-significant between age groups thereafter. These results are similar to the literature, which found differences between younger and older footballers [46], though presented here are results for each age group. These can be used as reference values for researchers and coaches who are using the same protocol and technology. Our correlation analysis found only a few low correlating factors to postural stability, and only foot length in unilateral postural stability was a factor related to foot morphology. This may be due to factors associated with postural stability beyond the scope of this research paper, such as maturation status [47] and strength of the trunk and lower limb [48]. The negligible correlations indicate that foot specific exercises should only be part of a multifaceted program to improve postural stability in footballers, as shown by multimodal interventions that lead to increased performance and decreased injury with better postural stability results [48, 49].

There are limitations to this research. These measures were taken with the participant in a static posture, and were not an analysis of dynamic measurements, which are usual practice in foot assessment [50]. An issue with 3D shapes and dynamic measurements is capturing the varying 3D shape over time, i.e., analysing a four-dimensional (4D) foot shape. Recent research has been promising in this area, and it is hoped that it continues to develop so foot morphology during gait can be compared to a general population for abnormalities, with footwear and treatment considerations from a functional perspective [51, 52]. The use of 4D foot morphology assessment would also be beneficial for longitudinal analysis to assess if the foot develops as described here. Future research could compare 3D foot shape with injury and/or pain development in participants to discover if any foot morphological features are associated with risk of injury or pain. This could inform cut-offs and abnormal ranges for 3D foot morphology for clinicians, and work similar to Stanković et al. for detecting abnormalities may streamline and standardise clinical assessment and treatment [51, 25].

This research only assessed male footballers and should be repeated in females, as there is less literature available on postural stability and foot morphology in this population [53, 54]. Further comparison to non-sport playing controls would be beneficial to determine the role of sport-playing on foot morphology and postural stability [45, 55]. Ethnicity was not gathered and should be a part of future research studies as there can be significant changes between different ethnical groups [56]. The clinical assessments used in this study were adapted for the 3D scans. They should be compared and validated compared to their 2D clinical equivalents. FPI-6 and Navicular Height measures were not used for this retrospective study due to their requirement of palpation for best practice [16, 57].

However, despite these limitations, there are clear practical applications from the results. Laser scanning enhances its value to clinicians when combined with SSM techniques, as the principal component analysis reduces the 3D foot into different shape features for analysis. This would normally require multiple assessments in the clinic, which would be time-consuming for each foot in comparison to a single scan. They are also not as reliable in replicating the 3D nature of the foot morphology as this study found. This becomes particularly important in assessment of adolescents.

Tracking of progression of 3D foot morphology in adolescence could identify those with abnormal morphology for potential treatment before pain and further deformity occur, which is preferable over a wait and see approach [19]. This identification can be personalised as the participant’s 3D foot can be compared to the population of the same age group with similar characteristics, i.e., male and football playing. The breakdown into the shape features can then identify where their foot significantly differs compared to the usual population, i.e., a significantly wider foot, flatter foot, etc., which can then lead to specific recommendations to the player, i.e., further dynamic and/or medical assessment, boot modification, exercise consideration. The larger datasets gathered, the reduced variability in the spread of the population across a shape feature, and the more accurate the clinician can be in determining what lies outside the normal variance for a given population for a given shape feature, i.e., determining an ‘abnormality’. This makes for precise, personalised care for the participant when examining the 3D foot shape.

As previously mentioned, foot assessment is warranted in youth footballers as there is repetitive loading on the foot-and-ankle complex, which could lead to overload from altered foot morphology [6, 9, 12]. We found significant differences in tibial rotation relative to the foot and foot arch morphology, which may led to significant changes in loading of the foot–ankle complex, which could predispose to pain and injury. As part of preseason assessment, the 3D foot scan could be analysed for these foot morphologies, with recommendations made for those in abnormal ranges for the upcoming season. Those in abnormal ranges could then be scanned more regularly to ascertain treatment or intervention benefit, with comparison to the population and their previous scans for an objective, personalised assessment. With the addition of comparison to postural stability measures, if an abnormality was found that was significantly related to poorer postural stability performance, it could be recommended to add postural stability exercises for that abnormal foot morphology. Future research and practice can expand on this by comparing 3D foot morphology to gait analysis, usual and sport-specific, for more movement-specific recommendations if altered loading is connected to a particular 3D foot morphology. Further, examining whether ‘abnormal’ ranges are then linked prospectively to injury and/or pain would further refine and personalise recommendations from 3D foot assessment.

## Conclusions

Using statistical shape modelling techniques on 3D foot scans, age groups were found to significantly differ in principal components (Modes) that described foot length, foot arch, and tibia rotation relative to the foot. The latter two did not follow a linear pattern towards a classic foot presentation at adulthood. Two usual clinical assessments (Clarke’s Angle and Resting Calcaneal Stance Position) were significantly correlated to and predicted the mode that specifies Arch Height (Mode 2). However, they were only low-to-moderately correlated, indicating that these 2D measures do not capture the complete picture of 3D foot morphology in flat or high arched feet. In turn, foot morphology was only lowly correlated with postural stability measurements, confirming that strategies to improve postural stability need to be multifaceted in their approach.

## Figures and Tables

**Figure:**
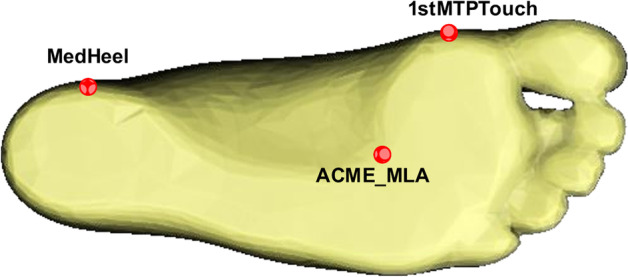
**Fig. 5** Three points are placed on the foot for the calculation of Clarke’s Angle. 1^st^MTPTouch: The most plantar aspect of the 1^st^ metatarsophalangeal joint. ACME_MLA: The acme of the medial longitudinal arch cavity. MedHeel: The most medial, plantar aspect of the heel

**Figure:**
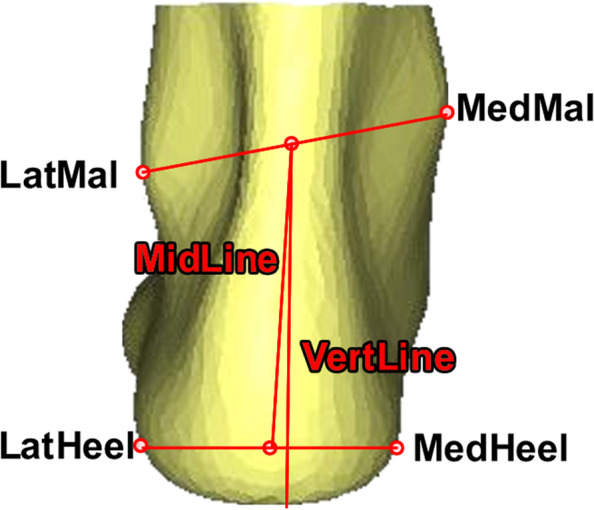
**Fig. 6 **Four points are placed on the foot for the calculation of Resting Calcaneal Stance Position (RCSP). LatMal: The centre, most prominent point of the distal lateral malleolus. MedMal: The centre, most prominent point of the distal medial malleolus. MedHeel: The most medial, plantar aspect of the heel. LatHeel: The most lateral, plantar aspect of the heel. Lines connected the points of the malleoli (MalLine) and the points of the heel (HeelLine), with midpoints marked on both lines. A line connecting the midpoints (MidLine) and a vertical line through the malleoli midpoint (VertLine) then created the RCSP angle

**Figure:**
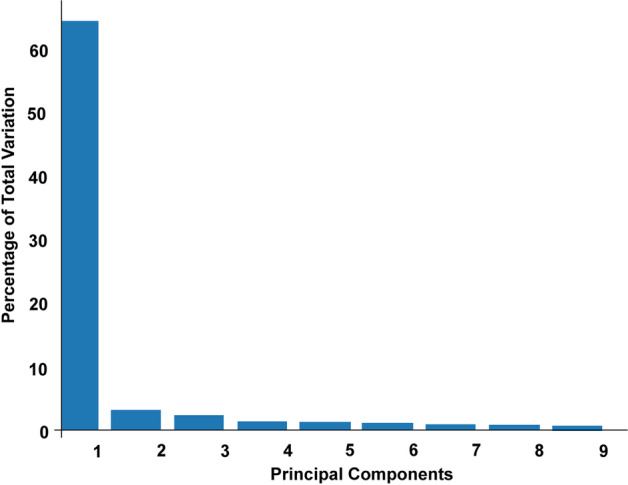
**Fig. 7** The nine modes of the statistical shape model that explain the most variation of foot shape, and the percentage variation they each explain

## Data Availability

The datasets generated and/or analysed during the current study are not publicly available due to the privacy of participants but anonymised data are available from the corresponding author on reasonable request.

## Abbreviations

2D: Two-dimensional
3D: Three-dimensional
4D: Four-dimensional
SSM: Statistical shape model
SD: Standard deviation
CA: Clarke’s Angle
RCSP: Resting Calcaneal Stance Position
BOE: Bilateral Open Eyes
BCE: Bilateral Closed Eyes
UOE: Unilateral Open Eyes
